# Bupivacaine 0.5 % versus articaine 4 % for the removal of lower third molars. A crossover randomized controlled trial

**DOI:** 10.4317/medoral.17628

**Published:** 2011-12-06

**Authors:** Manuel Sancho-Puchades, Miguel A. Vílchez-Pérez, Eduard Valmaseda-Castellón, Jordi Paredes-García, Leonardo Berini-Aytés, Cosme Gay-Escoda

**Affiliations:** 1 DDS. Fellow of Oral Surgery and Implantology, School of Dentistry, University of Barcelona (Spain); 2 PhD. Professor of Oral and Maxillofacial Surgery, Master’s Degree Program in Oral Surgery and Implantology, School of Dentistry, University of Barcelona, Barcelona, Spain. Researcher of the IDIBELL Institute; 3 DDS, MD. Professor of the Master’s Degree Program in Oral Surgery and Implantology, School of Dentistry, University of Barcelona, Barcelona, Spain. Researcher of the IDIBELL Institute; 4 DDS, MD, PhD. Dean, Professor of Oral and Maxillofacial Surgery, Master’s Degree Program in Oral Surgery and Implantology, School of Dentistry, University of Barcelona, Barcelona, Spain. Researcher of the IDIBELL Institute; 5 DDS, MD, PhD. Chairman and Professor of Oral and Maxillofacial Surgery. Director of the Master of Oral Surgery and Implantology. School of Dentistry of the University of Barcelona. Coordinator/Researcher of the IDIBELL Institute. Head of the Oral and Maxillofacial Surgery Department of the Teknon Medical Center, Barcelona (Spain)

## Abstract

Objective: To compare the anesthetic action of 0.5% bupivacaine in relation to 4% articaine, both with 1:200,000 epinephrine, in the surgical removal of lower third molars. As a secondary objective hemodynamic changes using both anesthetics were analyzed. Study Design: Triple-blind crossover randomized clinical trial. Eighteen patients underwent bilateral removal of impacted lower third molars using 0.5% bupivacaine or 4% articaine in two different appointments. Preoperative, intraoperative and postoperative variables were recorded. Differences were assessed with McNemar tests and repeated measures ANOVA tests. Results: Both solutions exhibited similar latency times and intraoperative efficacy. Statistical significant lower pain levels were observed with bupivacaine between the fifth (p=0.011) and the ninth (p=0.007) postoperative hours. Bupivacaine provided significantly longer lasting soft tissue anesthesia (p<0.05). Systolic blood pressure and heart rate values were significantly higher with articaine. Conclusions: Bupivacaine could be a valid alternative to articaine especially due to its early postoperative pain prevention ability.

** Key words:**Bupivacaine, articaine, third molar, anesthesia, postoperative pain.

## Introduction

Profuse scientific literature exists addressing different aspects of third molar treatment such as indications for extraction (1-3), complications (4), as well as multiple studies comparing effectiveness of different treatments protocols to control postoperative pain or edema (5,6). Third molar extractions have proven to be a suitable model to compare treatment approaches in a randomized controlled design (7). The ease to find individuals needing to undergo two symmetrical surgeries and the fact that patients act as their own control make this model very popular. This split-mouth design reduces possible research bias by avoiding physiological and psychological differences between tested individuals (8).

Local anesthetics (LA) have been broadly compared using this model (9-18). A variety of LA have been developed to satisfy specific requirements of different clinical procedures (19). Articaine is a common LA used in oral surgery. It belongs to the amide group of LA and has a fast onset and an adequate duration with little side effects (20). Even though it is considered a long lasting anesthetic there are others, such as bupivacaine, etidocaine or ropivacaine, with more extended anesthetic effects. Bupivacaine is often chosen in prolonged operations due to its extensive anesthetic period (13,17). Moreover, some authors have attributed it the ability to attain longer postoperative analgesic periods, reducing analgesic requirements in the early postoperative hours when the maximum pain intensity is reached (10,21,22). This feature is of major importance since one of the patient’s main concerns when undergoing a surgical procedure is the onset of postoperative pain (23).

Due to the clinical relevance of bupivacaine’s extended postoperative analgesic effect further analysis is needed. To our knowledge only a couple of clinical trials have compared bupivacaine with articaine for lower third molar removal. (15,24) Therefore, the aim of this study was to compare the intraoperative and postoperative anesthetic behavior of 0.5% bupivacaine in relation to 4% articaine, both with 1:200,000 epinephrine, in the surgical removal of symmetrically impacted lower third molars. As a secondary objective hemodynamic changes observed during the different surgical phases using both anesthetics were analyzed.

## Material and Methods

All patients provided written informed consent during the recruitment period of the study. The protocol of this study was approved by the Ethics Committee of the Dental School of the University of Barcelona. Calculation of sample size was performed using G*power 3.1.0 software, assuming α error =0.05, power= 95% and estimated effect size =0.4. Sixteen patients were needed.

To compensate for possible losses, study population consisted of 20 individuals with symmetrically positioned full bony impacted lower third molars, recruited from 25 eligible subjects. Eligibility criteria included ASA I or II patients, aged between 18 and 40 years, who presented bilateral impacted lower third molars, which required for their removal flap elevation, bone removal and tooth sectioning. Exclusion criteria included allergy to local anesthetics or any other medication, pregnancy or current lactation, heart rate > 110 bpm. or < 60 bpm., systolic arterial pressure > 150 mm Hg or < 100 mm Hg, diastolic arterial pressure > 100 mm Hg or < 60 mm Hg, oxygen saturation < 96%, pain, swelling or infectious signs associated to the third molar site immediately before surgery, any drug intake during the previous 15 days to the surgery, and surgeries lasting less than 15 minutes or longer than 45 minutes.

The study design comprised a triple-blind scheme. All anesthetic car pules were equally manufactured and were encoded. The patient, the surgeon and the statistician who performed the data analysis did not know which anesthetic solution had been used. Each patient required a similar surgical treatment for the removal of both inferior third molars. The extraction's were carried out at two different appointments, with at least a month of wash out period. The starting time of every surgery was 9:00 am. All surgeries were performed by the same surgeon and monitored by the same person.

The anesthetic technique chosen was a regional block of the inferior alveolar nerve at the mandibular foramen level with a direct technique (18), using a UnijectTM syringe (Becton&Dickinson, New Jersey, USA) with a 35 mm long and 27G Monoprotect XL® needle (Inibsa, Barcelona, Spain). Approximately 1.3 cc of the solution were deposited close to the mandibular foramen to anesthetize the inferior alveolar nerve and the remaining 0.5 cc were infiltrated while extracting the needle in order to anesthetize the lingual nerve. Next, 0.9 cc of the anesthetic solution was infiltrated in the buccal mucosa around the first and second lower molars to guarantee anesthesia and hemostasis of the site. Ten minutes after the anesthesia was delivered, thermal sensibility of the homolateral lower second molar was evaluated positioning a cotton pellet impregnated in tetrafluoroethane on the buccal aspect of the tooth. If the patient felt thermal stimulation, the anesthetic regional block was repeated and second molar pulpal sensibility was reevaluated 10 minutes after. Additional amounts of anesthetic were administered during the surgery if the patient complained about feeling pain. The surgical field and all surgical materials were sterile. The surgical technique was similar to that described by Leonard (25). Patients remained at the clinic for the first postoperative hour and were discharged if no complications arose.

Every patient received a leaflet where the postoperative instructions were described. Patients were prescribed 750 mg of amoxicillin (Amoxicilina Normon EFG 750 mg, Normon, Madrid, Spain) and 600 mg of ibuprofen (Ibuprofen Normon EFG 600 mg, Normon, Madrid, Spain) that had to be taken at 9:00 hours (just before surgery), 15:00 hours and 23:00 hours for 4 days. Patients were instructed to rinse with 0.12% chlorhexidine digluconate (Clorhexidina Lacer 0.12%, Lacer, Barcelona, Spain) 3 times a day for 15 days, starting the day after surgery. Metamizol was prescribed as a rescue medication (Metamizol Magnésico Normon EFG 575 mg, Normon, Madrid, Spain). Patients had to record the date and time at which the rescue medication was taken.

The following data were collected:

1. Onset of anesthetic action (in minutes) determined by: 1) the loss of pulpal sensibility assessed by a negative response to thermal stimuli after positioning a cotton pellet impregnated with tetrafluoroethane on the buccal aspect of the homolateral lower second molar and 2) loss of homolateral retromolar trigone mucosa, lip and tongue sensibility to pricking.

2. Total volume of anesthetic solution used during surgery and need of additional anesthetic infiltration's (time, volume, and anesthetic technique used for reanesthesia).

3. Duration of surgery after anesthetic administration (in minutes), which corresponds to the period between the first incision until placement of the last suture.

4. Adverse reactions during surgery or during the first postoperative week.

5. Intraoperative global pain judged by the patient and by the surgeon at the end of surgery in a 5-point scale (no pain, light pain, moderate pain, strong pain or unbearable pain).

6. Duration of postoperative anesthesia, represented by the lack of sensibility of the lower lip and the tongue. Patients recorded the moment at which they noticed the initial recovery of lip and tongue sensibility and the time at which lip and tongue sensibility had totally returned to normality.

7. Subjective pain evaluation, with the aid of a 100-mm-length visual analogue scale (VAS), with a 0 anchored by “no pain” and a 100 anchored by “worst pain imaginable”. Subjects recorded the intensity of postoperative pain at 2 hour intervals starting the surgery day at 10 am until 10 pm (10 am, 12 am, 2 pm, 4 pm, 6 pm, 8 pm, 10 pm), and during de second, third and fourth postoperative days only at 10 am and 10 pm.

8. Amount of rescue analgesic medication (magnesic metamizol) needed during the first 4 postoperative days.

9. Systolic and diastolic arterial pressure, heart rate and oxygen saturation before surgery, one minute after anesthetic infiltration, at tissue incision, at start of bone removal, and after suturing. All measurements were collected using the same monitoring instrument (Guardian BPM-730 M, Megos Sonmedica, Barcelona, Spain) in a noninvasive manner.

Paired t-tests were used to compare duration of surgeries and time to onset. Continuous variables were analyzed using repeated measures ANOVA. Differences between anesthetics for categorical variables were assessed with the McNemar test. Statistical significance was established at p<0.05. The results were presented as the mean ± standard deviation (SD).

## Results

Twenty patients were included in the study. Two of the participants were withdrawn from the study because they did not attend the second surgical appointment, so the final sample included eighteen patients (7 men and 11 women), with a mean age of 23.8 years (SD 5.0 years; range 18 to 35 years). No adverse reaction related to the anesthetic agent was detected during surgery or reported by any patient during the postoperative course.

The onset of action for articaine and bupivacaine as referred to lip and tongue numbness was similar (p=0.789), with a mean of 1.9 minutes (SD 1.2 minutes) and 1.8 minutes (SD 1.2 minutes) respectively.

Similarly, no significant differences were observed between both groups regarding number or volume of reanesthesia ( Table 1). Intraoperative reanesthesias were administered by means of intraligamentous infiltration, injecting 0.2 cc per infiltration. When a troncal reanesthesia was needed, 1.8 cc of anesthetic solution was injected. In six patients no additional anesthetic infiltrations were needed during any of the two surgical appointments. Eight patients needed supplementary infiltrations in both surgeries, while four patients needed reanesthesia only with one of the two solutions (3 with bupivacaine and 1 with articaine). No statistically significant differences were observed among solutions in this group of patients (McNemar test p=0.625).

**Table 1:**
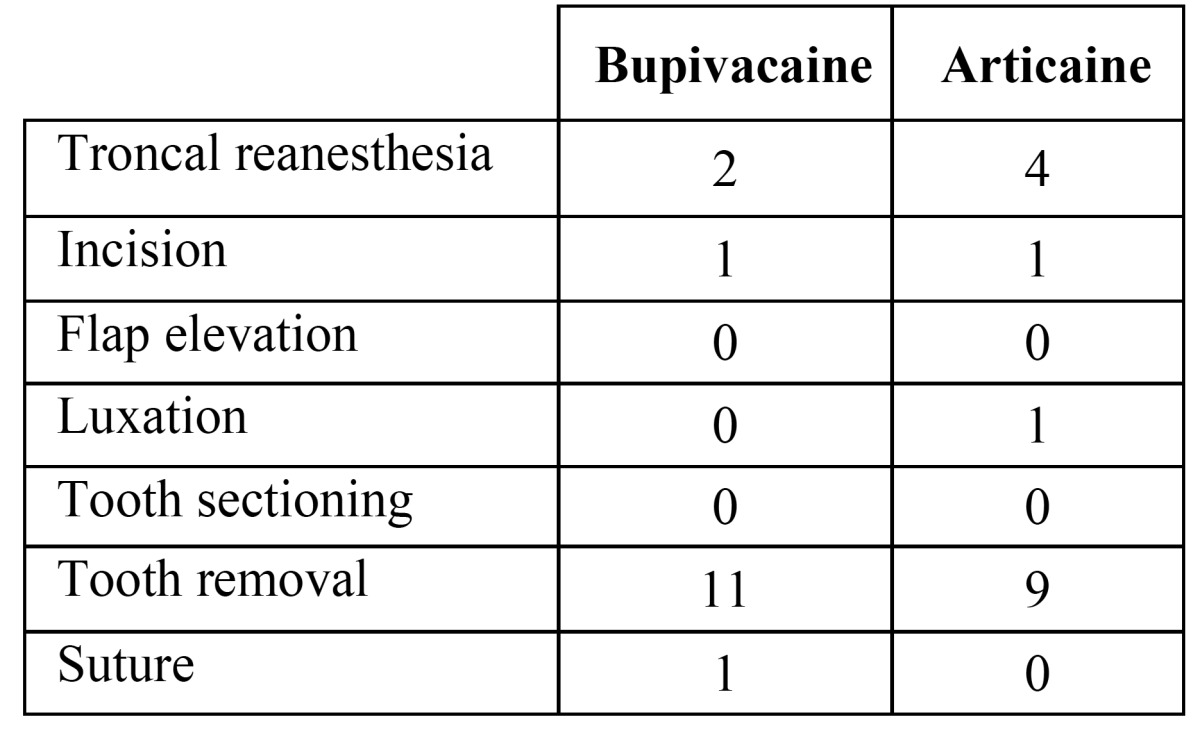
Number of additional anesthetic administrations. There were no significant differences between solutions (p>0.05).

Duration of surgery was also similar (p=0.185) with a mean of 23.7 minutes (SD 8.1 minutes) for bupivacaine vs. mean of 20.9 minutes (SD 4.5 minutes) for articaine. There were no statistically significant differences between solutions in pain perception, both interpreted by the surgeon or expressed by the patient ( Table 2). However, surgeons tended to underestimate patients’ pain sensation (in 9 tooth extractions using bupivacaine the patient rated greater pain scores than that rated by the surgeon, and in no case did the surgeon rate higher pain scores than the patient). This underestimation was significant with bupivacaine (McNemar test p=0.029), but not with articaine (McNemar test p=0.149).

**Table 2:**
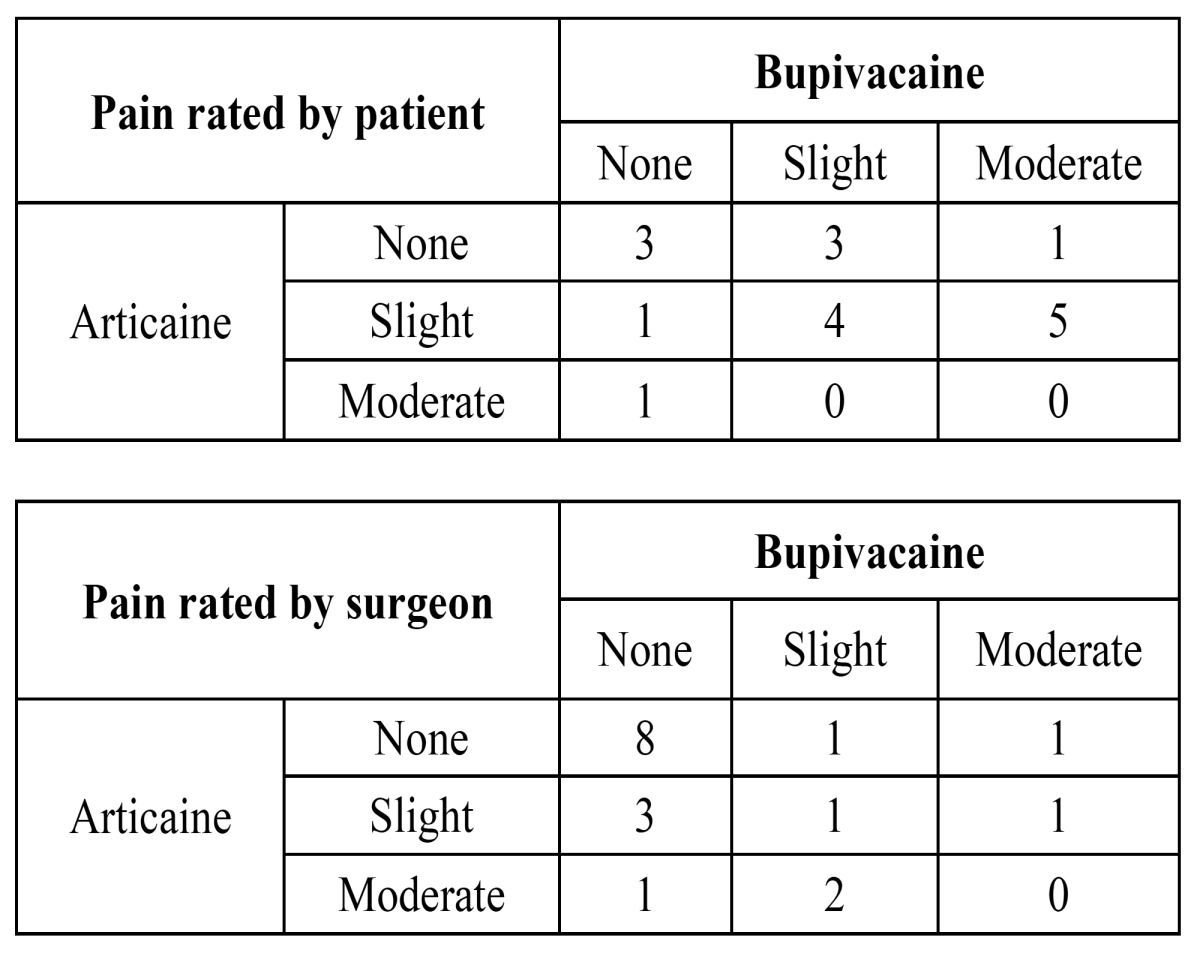
Intraoperative global pain judged by the patient and by the surgeon. Figures represent number of patients. Categories “strong” and “unbearable pain” were not marked by any patient or surgeon in any procedure. Nine patients rated pain higher with bupivacaine than with articaine (shadowed cells, upper table); only 2 patients rated pain higher with articaine (p=0.112). Conversely, the surgeon rated pain perception higher in 6 cases with articaine (shadowed cells, lower table) and in 3 cases higher with bupivacaine (p=0.721).

**Figure 1:**
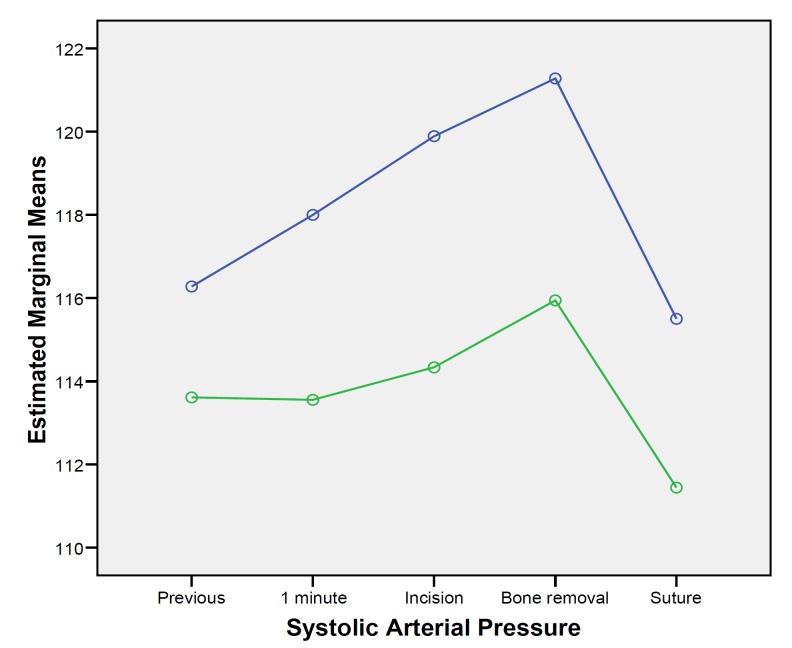
Systolic arterial pressure changes over surgery. (Articaine: blue line, Bupivacaine: green line).

**Figure 2:**
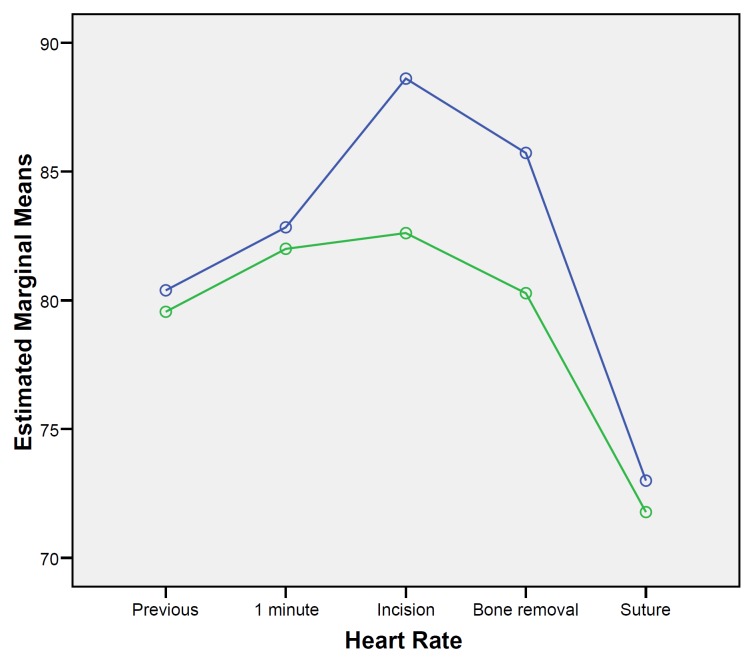
Heart rate changes over surgery. (Articaine: blue line, Bupivacaine: green line).

When considering hemodynamic changes through surgery between groups, statistically significant higher levels of systolic blood pressure were observed in the articaine group (F=7.658; df=1; p=0.013). Systolic pressure varied significantly across time (F=3.0; df=4, p=0.024), gradually increasing form the baseline until the bone removal measurement and descending again at suture placement (Fig. 1). However, this variation across time was similar for both anesthetic solutions (F=0.377; df=2.366; p=0.723). Diastolic blood pressure was similar between groups (F=1.051; df=1; p=0.320), with no significant changes across time (F=2.482; df=2.268; p=0.090). Similar results were recorded considering oxygen saturation, where values did not differ between groups (F=1.831; df=1; p=0.194) and no significant changes occurred through time (F=1.666; df=2.288; p=0.199). Conversely, heart rate values varied significantly between groups across time (F=2.733; df=4; p=0.036). Higher heart rate levels were observed in the articaine group at tissue incision and bone removal (Fig. 2).

Bupivacaine provided significantly longer lasting soft tissue anesthesia than articaine ( Table 3).

**Table 3:**
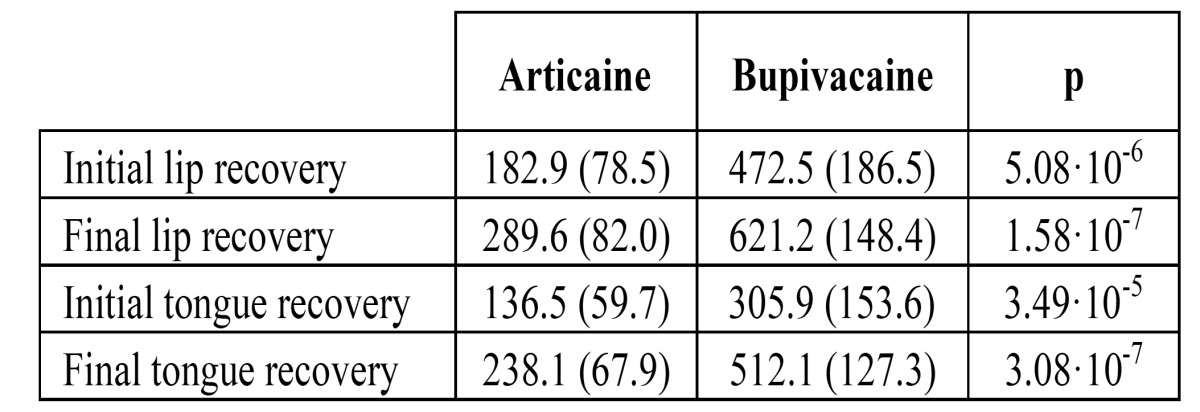
Mean duration (min) of soft tissue anesthesia (SD between parentheses). Articaine and bupivacaine were compared with a paired t-test.

**Figure 3:**
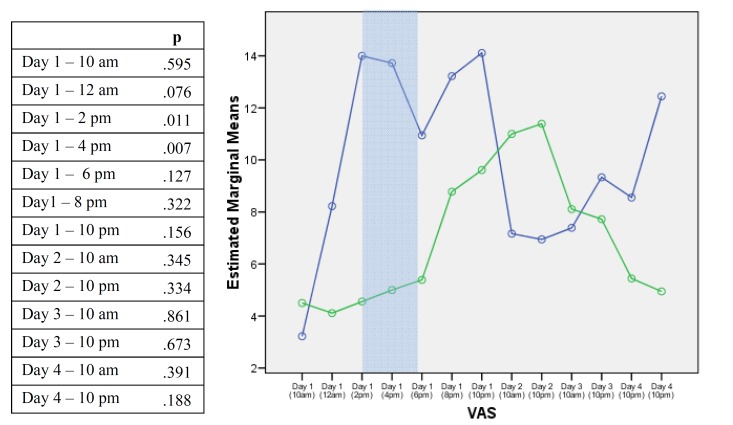
P values corresponding to differences between visual analogue scale scores with both anesthetic solutions at different times. The blue line corresponds to articaine and the green line to bupivacaine. Differences were statistically significant on day 1 at 2:00 pm and 4:00 pm (shadowed cells).

The postoperative VAS of pain varied significantly across time (F=2.114; df=12, p=0.017). Interestingly, the bupivacaine group had lower pain scores during day 1, being statistically significant at 2:00 PM (p=0.011) and 4:00 PM (p=0.007) (Fig. 3). No statistically significant differences were found concerning the total intake of rescue analgesics during the first four postoperative days ( Table 4).

**Table 4:**
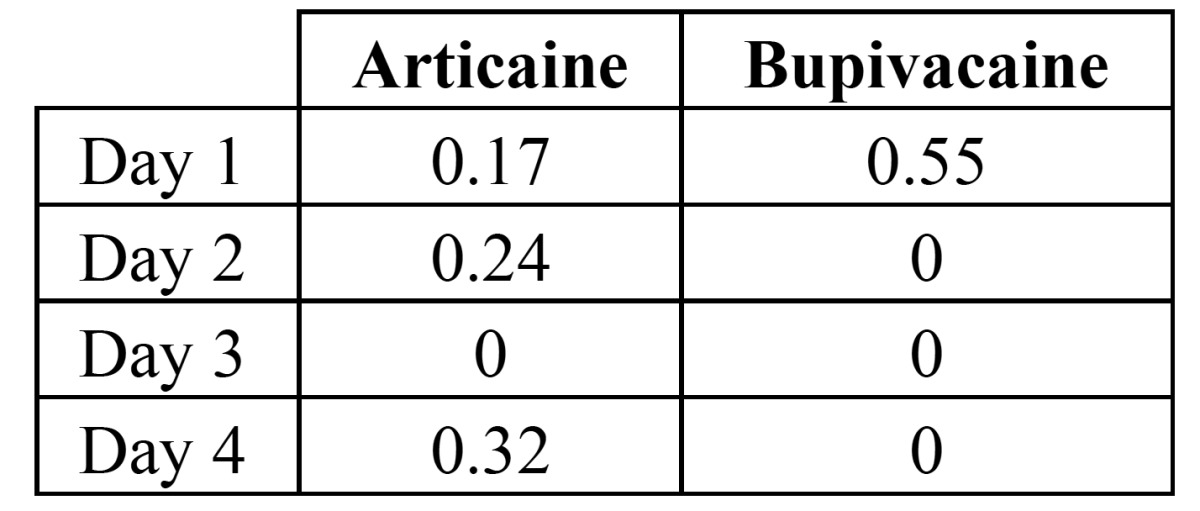
Mean number of magnetic metamizol tablets per day. There were no significant differences between solutions (p>0.05).

## Discussion

Our results suggest that 4% articaine and 0.5% bupivacaine, both with 1:200,000 epinephrine, have similar clinical efficacy in third molar surgery. Time to onset, need of additional anesthetic administration, intraoperative pain and hemodynamic effects were similar for both anesthetic solutions. The main difference between both solutions was the anesthetic effect duration; granting bupivacaine longer anesthetic periods, thus reducing early postoperative pain.

Even though a triple blind scheme was intended, the statistical analyzer’s blinding was jeopardized as a result of his awareness of the differences in anesthetic duration between local anesthetics. Nevertheless, the analyzer had no commercial compromise and the strategy for data analysis had been defined before the results were available.

In this study, no statistically significant differences on time to onset of action were observed between both solutions. Other studies have reported faster onsets with articaine, claiming lower pKa values (7.8) than bupivacaine (8.1) (15,24). Our results suggest that latency time is influenced by other factors besides pKa values. The anesthetic technique is probably an important factor, especially in the inferior alveolar nerve block, were the anesthetic solution is placed as close as possible to the inferior alveolar nerve, thus minimizing the need of dissemination of the anesthetic solution. Standardization of this troncal block is difficult as a result of anatomical variations among patients, differences between surgeons (right or left-handed, experience, etc.) and intraoperative circumstances (patient cooperation, operated side, etc.). Thus, we believe pKa values influence, but do not determine, onset of action, allowing similar and even shorter latency times for bupivacaine when compared to articaine solutions.

Need of additional anesthetic administration was similar for both solutions, however unexpectedly frequent. In the study by Gregorio et al. (15), with a similar design to ours, 14% of the patients required complementary anesthetic infiltration during the surgeries in which bupivacaine solution was used, while only 2% did so when articaine was administered. Conversely, a recent clinical trial with identical design demonstrated that the percentage of additional anesthesias was higher for articaine (47.4%) compared to bupivacaine (31.6%). In our study, 61% of the patients receiving bupivacaine and 50% receiving articaine requested for additional infiltration, especially with tooth avulsion maneuvers. The fact that patients did not perceive pain during tooth sectioning or luxation but did so during tooth avulsion maneuvers probably implies that patients could have misinterpreted traction forces as pain, making the blinded clinician administer more anesthesia. Supporting this idea, most of the patients who needed to be reanesthetized (8 out of 12 patients) required it during both surgeries, in other words, for both anesthetic solutions. This could suggest that the need for reanesthesia is more related to the patient than to the anesthetic solution itself.

When surveying the patients on intraoperative anesthetic efficacy no statistically significant differences were found between solutions. Nonetheless, 9 patients rated their intraoperative pain awareness higher with bupivacaine, were as only 2 did so with articaine. These results could imply that bupivacaine performs slightly worse in controlling intraoperative pain, although these differences did not reach statistical significance. Intraoperative pain awareness has not been addressed directly in other studies, however indirect signs of incomplete anesthetic depth, such as need to administer additional anesthesia, could prove to some extent an inferior performance of bupivacaine as compared to articaine (15).

Interestingly, the results suggest that surgeons are unable to interpret precisely painful situations. Surgeon’s ratings on the patients’ pain experience underestimated those expressed by patients in 25% of the surgeries performed. This should make surgeons consider improving patient-operator communication, in order to increase patient satisfaction and provide better care.

An important issue when studying the application of long lasting anesthetics in surgery is their ability to reduce or delay postoperative pain. In our study statistical significant lower pain levels were observed with bupivacaine between the fifth and the ninth postoperative hours with respect to articaine. This time period extends from the point where the articaine solution starts to wear off and the bupivacaine is still acting, until the moment where the anesthetic action of the bupivacaine solution disappears. Several studies confirm the ability of bupivacaine to prolong the analgesic period for inferior alveolar nerve block anesthesia, providing analgesia during the first 8 to 12 hours, which is the period of maximum pain after third molar surgery (9,10,14,26). Nevertheless, other studies fail to prove this residual analgesic effect (15) and even have shown lower early postoperative pain values with articaine when compared to bupivacaine (24). Differences between studies most likely respond to methodological design dissimilarities. It is therefore necessary to perform further research using greater sample sizes and strict parameter recordings to try to answer this critical uncertainty.

Even if bupivacaine could grant milder early postoperative periods, its extended anesthetic effect may be a drawback for some patients since prolonged periods of soft tissue anesthesia can cause soft tissue trauma as well as eating or speaking disability. Rosenquist and Nystrom (27) reported that 34% of patients described prolonged soft tissue numbness caused by bupivacaine anesthesia as unpleasant. In a further study conducted by Rosenquist et al. (18) some patients preferred having some postoperative pain if lip sensibility was recovered earlier. Mean lip anesthesia duration for bupivacaine in our study was 621 minutes. Significant differences in duration of lip anesthesia have been reported in other studies using similar volumes of the same anesthetic solution (306 minutes (24), 315 minutes (15), 411 minutes (14), 586 minutes (28), 643 minutes (11). Information about this issue should be given to the patient, evaluating his preferences with regard to extended lip anesthesia. Advice should be given to avoid soft tissue trauma. In our study no patient reported any traumatism on cheeks or lips. A suggestion to minimize this nuisance could be using long lasting anesthetic solutions when surgeries are performed in the afternoons. This would permit the patient going to sleep with no pain, reducing the potential hours of discomfort.

Oxygen saturation, heart rate and blood pressure suffered variations during stressful steps of surgery, as reported by Alemany-Martinez et al. (29). No significant changes were observed between anesthetic solutions with respect to oxygen saturation or diastolic blood pressure. However, higher systolic blood pressure levels and higher heart rate values reported in the articaine group at soft tissue incision and bone removal could be interpreted as a body reactions to a hectic or painful situation attributable to a lower anesthetic depth level compared to that obtained with bupivacaine. Nevertheless, results should be interpreted with caution since this reading contradicts the fact that patient’s reported similar or even worse intraoperative pain when receiving bupivacaine.

Even though bupivacaine has an overall good safety profile, it could have severe adverse effects on the central nervous system and cardiovascular system (19,30). For this reason, routine aspiration before injection is more than mandatory. Blood concentrations of bupivacaine studied by Bouloux et al. (10) after an inferior alveolar nerve block suggest that oral tissues modify the pharmacokinetics of the drug, slowing its vascular absorption, therefore decreasing its the potential toxicity. The present study failed to detect any signs or symptoms of central nervous system or cardiovascular toxicity in any subject with either local anesthetic.

Within the limitations of this study, it can be concluded that anesthetic success in third molar surgery is similar with 4% articaine and 0.5% bupivacaine, both with 1:200,000 epinephrine. Both solutions exhibit similar latency times and intraoperative efficacy. Bupivacaine intraoperative pain control seemed slightly worse. However, bupivacaine reduced pain scores during the early postoperative period, when the maximum intensity of pain occurs. This extended anesthetic effect entailed, nevertheless, prolonged periods of soft tissue numbness, which may be a nuisance. No significant changes were observed between anesthetic solutions with respect to diastolic blood pressure or oxygen saturation, although systolic blood pressure and heart rate were higher with articaine during incision and bone removal. Therefore, bupivacaine seems a valid alternative to articaine, particularly in the prevention of early postoperative pain.
